# Transcriptional Profiles of California Sea Lion Peripheral NK and CD^+8^ T Cells Reflect Ecological Regionalization and Infection by Oncogenic Viruses

**DOI:** 10.3389/fimmu.2019.00413

**Published:** 2019-03-12

**Authors:** Ignacio Peñín, Mónica E. Figueroa-Cabañas, Fabiola Guerrero-de la Rosa, Luis A. Soto-García, Roberto Álvarez-Martínez, Adriana Flores-Morán, Karina Acevedo-Whitehouse

**Affiliations:** ^1^Unit for Basic and Applied Microbiology, School of Natural Sciences, Autonomous University of Queretaro, Santiago de Queretaro, Mexico; ^2^The Marine Mammal Center, Sausalito, CA, United States

**Keywords:** California sea lion, cytotoxicity, NK, CD^8+^ T cells, otarine gammaherpesvirus, sea lion papillomavirus

## Abstract

The California sea lion is one of the few wild mammals prone to develop cancer, particularly urogenital carcinoma (UGC), whose prevalence is currently estimated at 25% of dead adult sea lions stranded along the California coastline. Genetic factors, viruses and organochlorines have been identified as factors that increase the risk of occurrence of this pathology. Given that no cases of UGC have as yet been reported for the species along its distribution in Mexican waters, the potential relevance of contaminants for the development of urogenital carcinoma is highlighted even more as blubber levels of organochlorines are more than two orders of magnitude lower in the Gulf of California and Mexican Pacific than in California. *In vitro* studies have shown that organochlorines can modulate anti-viral and tumor-surveillance activities of NK and cytotoxic T-cells of marine mammals, but little is known about the activity of these effectors in live, free-living sea lions. Here, we examine leukocyte transcriptional profiles of free-ranging adult California sea lions for eight genes (Eomes, Granzyme B, Perforin, Ly49, STAT1, Tbx21, GATA3, and FoxP3) selected for their key role in anti-viral and tumor-surveillance, and investigate patterns of transcription that could be indicative of differences in ecological variables and exposure to two oncogenic viruses: sea lion type one gammaherpesvirus (OtHV-1) and sea lion papillomavirus type 1 (ZcPV-1) and systemic inflammation. We observed regional differences in the expression of genes related to Th1 responses and immune modulation, and detected clear patterns of differential regulation of gene expression in sea lions infected by genital papillomavirus compared to those infected by genital gammaherpesvirus or for simultaneous infections, similar to what is known about herpesvirus and papillomavirus infections in humans. Our study is a first approach to profile the transcriptional patterns of key immune effectors of free-ranging California sea lions and their association with ecological regions and oncogenic viruses. The observed results add insight to our understanding of immune competence of marine mammals, and may help elucidate the marked difference in the number of cases of urogenital carcinoma in sea lions from US waters and other areas of their distribution.

## Introduction

The California sea lion (*Zalophus californianus*) is one of the few wild mammals prone to develop cancer under natural conditions. Since the initial report in the early 80's, the prevalence of urogenital carcinoma has remained consistently high, with *post mortem* examination of dead individuals revealing a prevalence of up to 25% in adult sea lions necropsied after stranding along the California coast (1). The high incidence of such an aggressive and fatal pathology in a long-lived top predator of the coastal marine ecosystem warrants studies to increase our understanding of the factors that contribute to its occurrence.

As is the case for most cancers, sea lion urogenital carcinoma appears to be multifactorial, and various risk factors have been identified. These factors include an oncogenic genital gammaherpesvirus, named OtHV-1 (2, 3) and genetic components (4–6). Furthermore, high concentrations of organochlorines have been detected in the blubber of sea lions with urogenital carcinoma (7). This latter association is particularly relevant as studies conducted in laboratory animals have shown that organochlorines can induce carcinogenesis, either directly at high concentrations (8) or indirectly by modulating immune responses, particularly when exposure is low (9–11). *In vitro* experiments with different marine mammal cells have shown that organochlorines modulate NK and cytotoxic T-cell activity (12–14). Based on their known anti-viral and tumor surveillance activity (15–17), and the evidence of organochlorine-induced modulation, it is parsimonious to speculate that NK and cytotoxic T-cells play an important role in preventing the development of urogenital carcinoma in the California sea lion, and that these immune effectors are sensitive to extrinsic and intrinsic factors.

Despite its high prevalence in California, urogenital carcinoma has not been observed in sea lions inhabiting the Gulf of California, in spite of systematic surveys of the breeding colonies by researchers and park managers. However, pre-cancerous transformation of the genital epithelium, including binucleation and koilocytes, do appear to be relatively common in California sea lions from the Gulf of California (18). In humans, the presence of these cellular phenotypes is considered the first step toward carcinogenesis if the abnormal cells are not promptly detected and destroyed by tumor-surveillant and cytotoxic immune cells (19). Interestingly, compared to values reported for sea lions in California (7), blubber PCB levels are three orders of magnitude lower (20) in sea lions from the Gulf of California, and two orders of magnitude lower in sea lions from the Mexican North Pacific (21). This implies that there could be differences in NK and cytotoxic T-cell activity (12–14), which could, in turn, result in differences in oncogenesis.

Within the Gulf of California, 13 sea lion breeding colonies are spread along 177,000 km^2^, from the northernmost colony, Rocas Consag, located at less than 100 km from the Colorado River Delta, to the southernmost colony, Islotes, 29 km from the city of La Paz, in the tip of the peninsula of Baja California. Oceanographic and ecological differences among zones have led to regionalization of the Gulf of California, and colonies are grouped in four main regions (22), largely defined by upwelling and phytoplankton profiles (23) that influence the availability of resources (24, 25). Sea lion colonies vary per region in terms of population trends (26), genetic substructure (27, 28) and pathogen exposure (29). In terms of pollutants, there is a marked latitudinal gradient, with the northern region being more polluted than the southern region due to deposition from the Colorado River Delta (30).

Based on the species' genetic substructure in the Gulf of California, and on spatial differences in oceanographic and ecological factors, it is plausible to assume that California sea lions experience intra- and interregional differences in terms of their exposure to organochlorine pollutants and other extrinsic factors that could impact their tumor surveillance and cytotoxic capability, as well as induce chronic inflammation. Furthermore, as disposable energy in a region is limited by its productivity, prey availability, and feeding range (25), it is unlikely that a sea lion's investment of resources for immune activities will be independent of its environment. Some evidence for this phenomenon has already been reported for this species (31).

Lymphocyte development and activation are characterized by marked changes in gene expression (32). Thus, we hypothesized that transcription of key genes relevant to immune surveillance of tumors and cytotoxic responses of California sea lions differs spatially and is related to genital infection by two oncogenic viruses, sea lion type one gammaherpesvirus (OtHV-1) and sea lion papillomavirus type 1 (ZcPV-1), and systemic inflammation. To challenge our hypotheses, we examined transcription patterns of key genes expressed by NK, CD^8+^ T cells and by T regulatory cells in the blood of apparently healthy adult females sampled at breeding colonies within the Gulf of California and the Mexican North Pacific.

## Materials and Methods

### Collection of Samples

**Figure 1:**
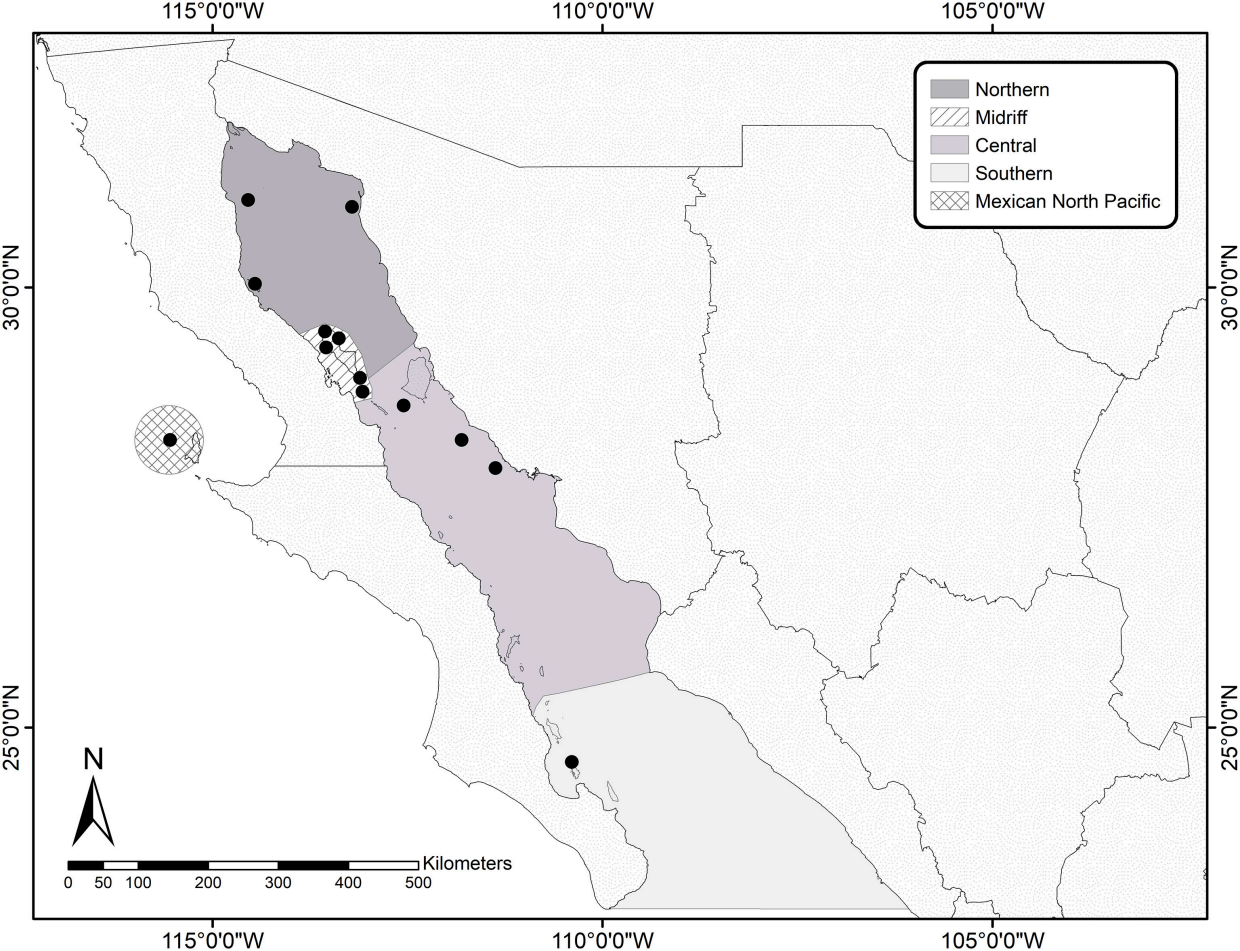
Location of the sampling sites within the Gulf of California and Mexican North Pacific. The map shows the ecological regionalization proposed by Stzeren and Aurioles (22) for the Gulf of California and the San Benito Archipelago on the west coast of the Baja California Peninsula, in the Mexican North Pacific. Black dots indicate the colonies where the sea lions were sampled.

During the summer of 2016 we visited 12 California sea lion breeding colonies in the Gulf of California. Based on the ecological regionalization proposed (22), we sampled sea lions in three colonies in the Northern region (Rocas Consag, San Jorge, and Lobos), three in the North-Central (Midriff) region (Granito, Cantiles, and Los Machos) five in the Central region (Partido, El Rasito, San Esteban, San Pedro Mártir, and San Pedro Nolasco), and one (Los Islotes) in the far South region (Figure 1).

Samples of peripheral blood were collected from 54 apparently healthy adult female California sea lions, which had been captured using hoop nets, and manually restrained before using inhaled anesthesia (Isoflurane). Once anesthetized, a trained veterinarian examined the sea lions in order to determine their general health, and to monitor their heart rate and respiratory frequency throughout the procedure, which lasted 10–12 min. All sea lions were considered to be in good body condition, with at least 20 mm of lateral blubber depth (skin fold thickness measured with calipers). Two 7-ml blood samples were collected from the caudal gluteal vein of each sea lion with vacuum tubes (Vacutainer), one of them coated with sodium heparin and one with EDTA. Genital epithelial swabs were also collected. Briefly, a sterile genital speculum was introduced to visualize the cervix and the cervical mucosa was scraped with a sterile cytobrush. The swabs were stored in a vial containing 96% ethanol and were protected from sunlight until staining in the laboratory. The blood samples were centrifuged using a clinical centrifuge (Clay Adams compact II, Daigger Scientific, USA) for 10 min at a fixed speed of 3,200 rpm within 3 h after collection. The buffy coat was separated using a sterile Pasteur pipette and stored immediately in cryogenic tubes prior to snap-freezing in liquid nitrogen, where kept until processing. EDTA-preserved blood was used to total and differential leukocyte counts. Based on the hematological information, we determined the neutrophil to lymphocyte ratio (NLR) as a measure of chronic systemic inflammation and stress (33), which has been shown to be a useful marker in the California sea lion (34). We also had equivalent samples from nine adult female sea lions, which had been captured during the summer of 2014 at a colony situated in the Archipelago of San Benito, in the Mexican North Pacific (see Figure 1). Sampling and processing of these samples was performed as described above. Adult males were not included in our study due to the difficulty and extremely high-risk of capturing and restraining these individuals in the field.

All procedures were approved by the Bioethics committee of the Universidad Autónoma de Queretaro (Mexico), and were conducted under permits SGPA/DGVS/11744/13 (for samples collected in 2014) and SGPA/DGVS/09004/15 (for samples collected in 2016) issued by the Secretariá de Medio Ambiente y Recursos Naturales through the Direccioń General de Vida Silvestre in Mexico.

### Relative Quantification of Gene Expression in Lymphoid Subpopulations

We extracted total RNA from the buffy coat samples using Trizol (Sigma-Aldrich, USA) as per the manufacturer's instructions. We treated the solubilized RNA with DNA-free™ DNA removal kit (Thermo Fisher Scientific, USA) as per the manufacturer's instructions. RNA integrity was assessed by electrophoresis in a 1% agarose gel stained with ethidium bromide and purity was determined by Nanodrop spectrophotometry (Qiagen, USA). Good quality samples were those that had no evidence of RNA degradation, showed clear 28S and 18S rRNA bands (see Figure S1), and whose A_260_/A_280_ ratio was between 1.75 and 2.10. Five of the RNA samples did not clearly show the two rRNA bands despite having a good A_260_/A_280_ ratio. In these cases, we re-extracted the RNA and reassessed the quality. In all of these cases, we were able to confirm that quality was adequate to proceed with reverse transcription (see Supplementary Material for more details).

Reverse transcription was performed for each sample with a QuantiTect Reverse Transcription Kit (Qiagen, USA) using 200 ng of RNA in 20 μL reactions. As per the manufacturer's instructions, the procedure included a 2 min. incubation at 42°C with gDNA wipeout buffer to further ensure the elimination of genomic DNA contamination. cDNA was frozen in aliquots to avoid repeated freeze-thaw cycles.

The levels of transcription of eight genes were assessed by real time quantitative PCR (RT-qPCR). The genes were selected *a priori* to represent the activity of different lymphocyte subpopulations and immune activities relevant to antiviral and antitumor activities (see Table 1 for genes and primers). RPS5 and HpRT were used as reference (housekeeping) genes as they are expressed in all nucleated cells, and we have previously shown their transcription levels to be stable in California sea lion peripheral white blood cells (35). In order to comply with the Minimum Information for Publication of Quantitative Real-Time PCR Experiments (MIQE) guidelines (36), we used a subset of 10 samples to run RT-qPCR to evaluate expression stability. This allowed us to confirm that both genes were appropriate to use as reference genes (see details and Figure S2). All primers were designed to span exon-exon junctions. All primer pairs were evaluated for their efficiency. The efficiency of the primers ranged between 90 and 112%, and the coefficient of determination (*R*^2^) was >0.95 (E and *R*^2^ results for all the primers used for RT-qPCR can be seen as Figures S3, S4).

**Table 1:** Genes selected as markers of NK and CD^8+^ cytotoxicity, Th2 responses and immunomodulation.

**Gene**	**Encoded protein and function**	**Primers (5′-3′)**
*Ly49*	Inhibitory receptor of NK cells.	F. TGTCAGAGAGGAAATGAAGGCA R. TGGCAAGTCTGTTTACATCCGT
*Granzyme B*	Cytotoxic serum protease that induces apoptosis of target cells. Exerts antiviral and antitumoral responses.	F. CACTCTGCAAGTAGTGAGGCT R. CAGCTGAATGGTGTGGTCGTA
*Perforin*	Glycoprotein responsible for forming pores in the cell membrane of target cells. Mediator required for apoptosis; Relevant for antiviral and antitumoral responses.	F. CCTGCTGCAGTTCTTCCAAC R. CTGGCACTGACCGACTGG
*Eomes*	Eomesodermin (T-box brain protein 2). Leads activation and differentiation of TCD^8+^; reflects antitumoral responses.	F. TCAGTCCTTCTCCCGGAGC R. GGTTGACCACCTTTCGTTCTG
*STAT-1*	Signal transducer and activator of transcription 1. Mediates responses to interferon and cytokines; induces antiviral state.	F. GGTGAACTGGACCCCAGTCT R. CTATGGGACCGCACCTTCAA
*Tbx21*	T cell associated transcription factor (Tbet). Member of the T-box family of transcription factors expressed in Th1 cells. Directs T-cell differentiation and represses Th2 responses.	F. GAGGCTGAGTTTCGAGCAGT R. AGTAGGACATGGTGGGTCCG
*GATA-3*	Trans-acting T-cell-specific transcription factor. Promotes secretion of anti-inflammatory cytokines by Th2 cells; inhibits the expression of IFNγ; suppresses differentiation of naïve T-helpers to Th1 cells. Expressed by T cells, NK cells and CD1-restricted NKT cells.	F. CATGACACGCTGGAGGACTT R. AGGGAGGTCATGTGTCTGGA
*FoxP3*	Transcription factor forkhead box protein P3. Anti-inflammatory and anti.apoptotic role. Shapes immune tolerance.	F. TGCAGTCTCTGGAACAGCAG R. TTTGGTCAGGGCCATCTTCC

RT-qPCR reactions contained 4 microliters of cDNA (1:4 to 1:16 of template generated by retrotranscription of 10 ng/μL of RNA), 0.15 μL of each primer (at 0.2 μM each), 5 μL SYBR® Green master mix (Thermo Fisher), and 0.7 μL of water to reach 10 μL of final volume. The reactions were run on a CFX Connect™ Real-Time PCR Detection System (BioRad, USA) as follows: 95°C for 15 min, followed by 40 cycles of 15 s at 95°C, 1 min at 55C (during which the plate was read) and 72°C for 1 min. The ending cycle was kept at 95°C for 15 s and a final step for the melting curve at 60 to 90°C (0.5°C increase and 15 s of wave length measurement for each temperature). Reaction specificity was monitored by melting curve analysis using a final data acquisition phase of 60 cycles of 65°C for 30 s. The threshold was established manually after amplification take-off. Optimal results were achieved when using 1:2–1:8 of cDNA. For each primer pair, a reaction mixture containing water, but no cDNA, was used as a non-template control (NTC) to monitor contamination and primer dimer formation; a no-reverse transcriptase (-RT) mixture was included as a control to monitor DNA contamination. Gene expression levels were determined by relative quantification (i.e., transcription of the target gene relative to the average of the reference genes) as per the ΔCt method (37). The data were considered reliable if the difference between replicates was below one cycle. If the reliability of the reference genes failed, the samples were rerun for all the primer pairs of a plate. Optimal results were achieved when using 1:2–1:8 of cDNA.

### Molecular Detection of Oncogenic Viruses

Genomic DNA was extracted from the ethanol-preserved genital swabs using a routine proteinase K digestion followed by a phenol-chloroform protocol and isopropanol precipitation. DNA was quantified and quality was assessed using a spectrophotometer (Nanodrop 2000, Thermo Fisher Scientific, USA). We amplified a 210 bp fragment of the DNA polymerase (Dpol) gene of OtVH-1 in genital swabs. Primers used were Dpol 697 5′- GCGGGAACGCAACTATATCCT and Dpol 65 5′-TCTTCGTCCAGTATCATTG; (38). For ZcPV-1, we used GenBank sequence NC_015325 (*Zalophus californianus* papillomavirus 1) as a reference to design a pair of primers that amplified a 450 bp fragment of gene L2 (ZcPV_F5957-ATACAGGACGGGGACATGG, ZcPV_R6495-TCATATTCCTCAGCGTGCCT).

All 12.5 μl PCR reactions were performed on an ABI 3100 thermal cycler (Applied Biosystems, Inc.) and were conducted in duplicate. Cycling conditions were 95°C for 15 min, 30 cycles of 94°C for 40 s, 52°C (OtHV-1) or 53°C (ZcPV-1) for 30 s and 72°C for 40 s, and a final extension step at 72°C for 10 min. A template free (template free reaction) was included with every PCR. Amplified products were electrophoresed on an ethidium bromide stained 1.8% agarose gel and visualized on a UV transilluminator. To ensure the amplified products were not the result of non-specific amplification, for each pathogen, two bands selected at random were gel excised, column purified (QiaQuick, Qiagen, USA), cloned and bi-directionally sequenced for confirmation. Each sequence was visually inspected and compared to those reported in GenBank (http://www.ncbi.nlm.nih.gov/genbank/).

### Statistical Analyses

Before beginning the analyses, we examined the normality and homoscedasticity of the relative level of transcription of each target gene with Shapiro and Bartlett tests, respectively. None of the gene expression levels showed deviation of normality or heteroscedasticity. We used Spearman correlations to identify relationships between gene transcripts. The *GATA3* to *Tbx21* (hereafter Ga/Tb) ratio, a measure of Th1/Th2 profile (39) deviated from expectations of normality and, based on a Cullen and Frey graph, appeared to follow a beta distribution. Goodness of fit was examined with a Kolmogorov test.

To challenge our hypotheses, we first built generalized linear models (GLM) for each of the genes, and the Ga/Tb ratio defining the error family as per the distribution of the error (40), and indicating the region where each sea lion was sampled as the explanatory variable. We next used independent GLMs to examine whether the transcription level of each gene and the Ga/Tb ratio was affected by the presence of OtHV-1, ZcPV-1, or simultaneous infection by both viruses in the genital epithelium, and whether gene transcription levels were influenced by systemic inflammation, as assessed by the NLR of the sea lions. For genes whose transcription levels varied among regions, we included this variable in the respective models. We used a top-down strategy to determine which variables explained a significant fraction of the data (40), and we examined the residual distribution by inspecting Q-Q plots, Cook distance and plots of the adjusted residuals *vs*. the obtained residuals to validate the model. As lower ΔCt values represent higher levels of expression, interpretation was made easier by using a negative transformation of the response variable (–log ΔCt). To account for differences in prevalence of OtVH-1, ZcPV-1 and simultaneous infection among regions, we built contingency tables and ran Fisher exact tests. In all cases we considered results statistically significant if the *p*-value was less than 0.05.

Our second approach was to analyze gene transcription profiles with no pre-defined regionalization to allow natural clustering of immune profiles. For this, data were subjected to hierarchical clustering to generate heat maps and dendrograms of the gene transcripts according to the degree of similarity of the transcriptional profiles. Multiple imputation chain equation was used to account for missing values. We first used the full set of target genes, as the “complete profile” of each individual. Next, we used the relative expression of genes representative of responses relevant to this study: (i) NK and CD^8+^ activity and differentiation (*Ly49, perforin, granzyme, STAT-1, Tbx21*, and *Eomes*), (ii) Th2 and immune modulation (*GATA3* and *FoxP3*), and (iii) Th1/Th2 ratio (*Tbx21* and *GATA3*). The number of natural clusters was determined according to a rarefaction curve and two dendrograms were initially created for each set of genes. Algorithms used were Ward.D2 and Average (41). Goodness of fit of the models was based on the cophenetic correlation coefficient, with an optimality value of ≥0.8. As values were lower than 0.8 for all models, we used an Euclidean approach to build the final dendrogram (42). All statistical analyses were conducted in R v3.4.2 (43) using core packages as well as *cluster, dendextend, factoextra, fitdistrplus, mice, prevalence* and *ggplot2*.

## Results

### NK and CD^8+^ Activity

Most of the transcription levels of the six genes involved with NK and CD^8+^ activity were significantly correlated to each other (Table 2), with correlation coefficients ranging from 0.75 to 0.31. The exceptions were *Ly49* and *perforin*, both of which were not correlated to *STAT-1*.

**Table 2:** Spearman's correlation coefficients of genes expressed by NK and CD^8+^ T cells.

	***Eomes***	***STAT-1***	***Tbx21***	***Granzyme B***	***Perforin***	***Ly49***
*Eomes*	–	0.55	0.83	0.63	0.70	0.36
*STAT-1*	0.0008	–	0.62	0.33	0.30	0.17
*Tbx21*	0.0000	0.0000	–	0.37	0.58	0.40
*Granzyme B*	0.0000	0.0452	0.0240	–	0.50	0.44
*Perforin*	0.0000	0.0880	0.0004	0.0021	–	0.34
*Ly49*	0.0412	0.2316	0.0028	0.0059	0.0477	–

*The bottom half of the table shows p-values*.

**Figure 2:**
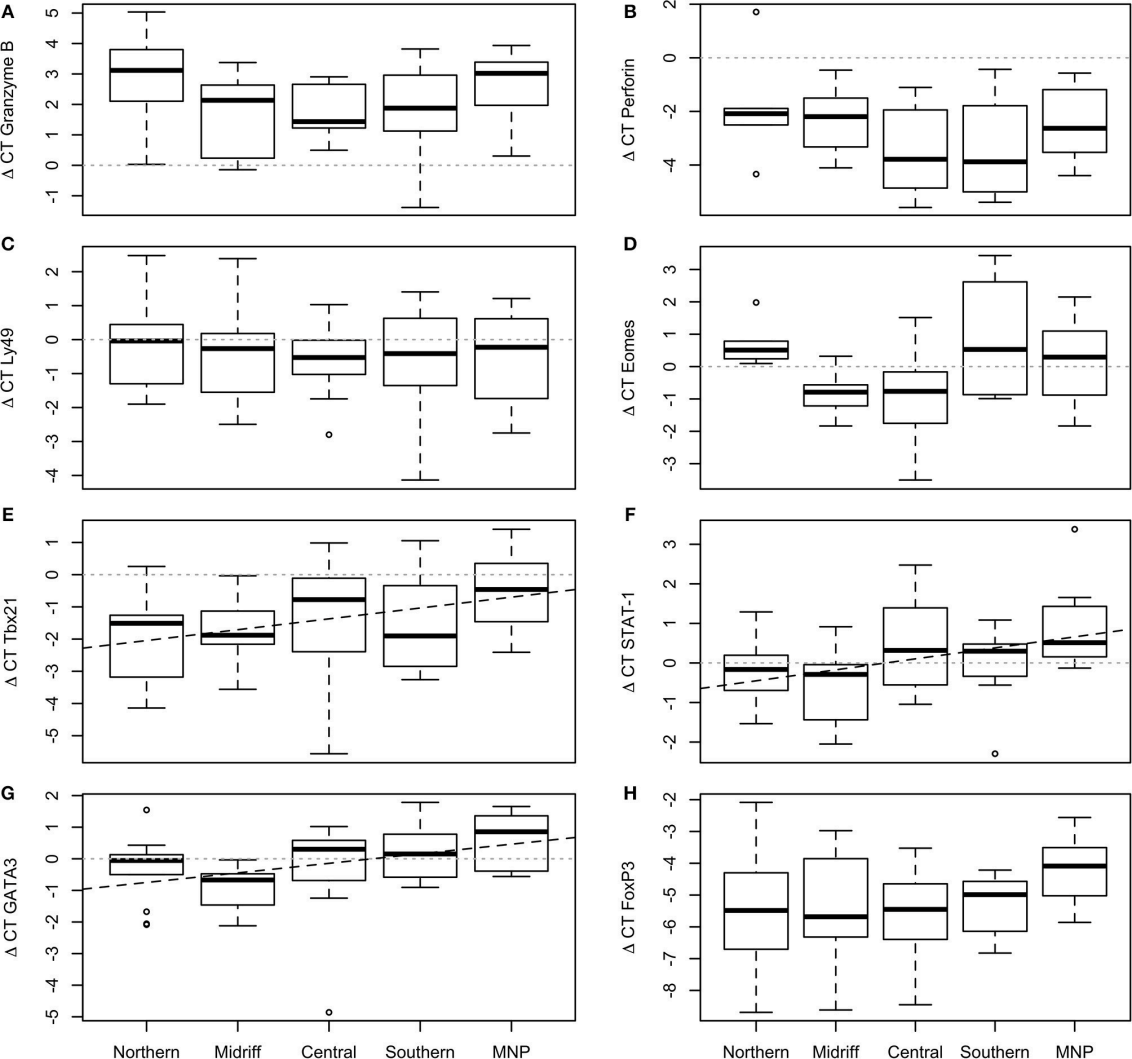
Transcription levels of selected genes in California sea lion blood sampled at different regions. **(A)** *Granzyme B*, **(B)** *Perforin*, **(C)** *Ly49*, **(D)** *Eomes*, **(E)** *Tbx21*, **(F)** *STAT-1*, **(G)** *GATA3*, **(H)** *FoxP3*. *Ly49, perforin, granzyme B* and *FoxP3* did not vary significantly among regions. *Eomes* was slightly downregulated in sea lions from the midriff and central regions, and upregulated in the northern and southern regions, and the Mexican North Pacific. *Tbx21, STAT-1* and *GATA3* increased from north to south within the Gulf of California and reached highest levels in the Mexican North Pacific. The plot shows the median (thick line), first and third quartile (box), and 95% confidence interval of the median (whiskers).

Transcription levels of *Ly49, perforin* and *granzyme B* did not vary significantly among regions [GLM; *Ly49*: *F*_(4, 49)_ = 0.21, *p* = 0.932, Figure 2C; *perforin*: *F*_(4, 31)_ = 1.44, *p* = 0.244, Figure 2B; *granzyme B*: *F*_(4, 34)_ = 1.26, *p* = 0.304; Figure 2A]. In nearly all sea lions, *granzyme B* was upregulated more than 2-fold with respect to the reference genes, while *perforin* showed the inverse pattern, being downregulated on average 2.73-fold; *Ly49* was slightly downregulated in all but a few individuals, with only a few sea lions showing upregulation.

Transcription of the three transcription factors involved with Th1 development varied among regions [GLM; *Eomes*: *F*_(4, 30)_ = 2.64, *p* = 0.049, Figure 2D; *Tbx21*: *F*_(4, 49)_ = 6.22, *p* = 0.016, Figure 2E; *STAT-1*: *F*_(4, 49)_ = 3.58, *p* = 0.012; Figure 2F]. *Eomes* was slightly downregulated (average: 0.93-fold) with respect to the reference genes in the majority of sea lions from colonies within the midriff and central regions, and upregulated in the northern and southern regions, as well as in the Mexican North Pacific (average: 0.38-fold). *Tbx21* was mostly downregulated, and levels increased across samples collected from north to south within the Gulf of California, reaching the highest values in animals from the Mexican North Pacific (Adj. *R*^2^ = 0.09, *df* = 52, *p* = 0.016; Figure 2E). In contrast to the other genes indicative of Th1 development, *STAT-1* was generally upregulated, and levels of transcription also exhibited a north to south trend within the Gulf of California, reaching the highest values in the sea lions from the Mexican North Pacific (Adj. *R*^2^ = 0.11, *df* = 52, *p* = 0.007; Figure 2F).

### Th2 and Immune Modulation

*GATA3* and *FoxP3* were significantly correlated (*r* = 0.39, *p* = 0.006). Transcription of *GATA3* varied significantly among regions [GLM; *F*_(4, 49)_ = 3.19, 0.017], with a north-to-south increasing gradient within the Gulf of California, and showing the highest levels of transcription in sea lions from the Mexican North Pacific (Adj *r*^2^ = 0.12, *df* = 52, *p* = 0.006; Figure 2G). *FoxP3* was consistently downregulated (average: 5.29-fold), with no significant differences among regions [GLM; *F*_(4, 44)_ = 1.35 *p* = 0.268; Figure 2H].

### Th1/Th2 Ratio

There was a wide variation in Ga/Tb, with values ranging to 45.29 to −1.49 (mean = 1.64), and there was no evidence of differences among regions [GLM; *F*_(4, 49)_ = 0.81, *p* = 0.528].

### Patterns of Gene Transcription Levels Using no Pre-defined Regionalization

**Figure 3:**
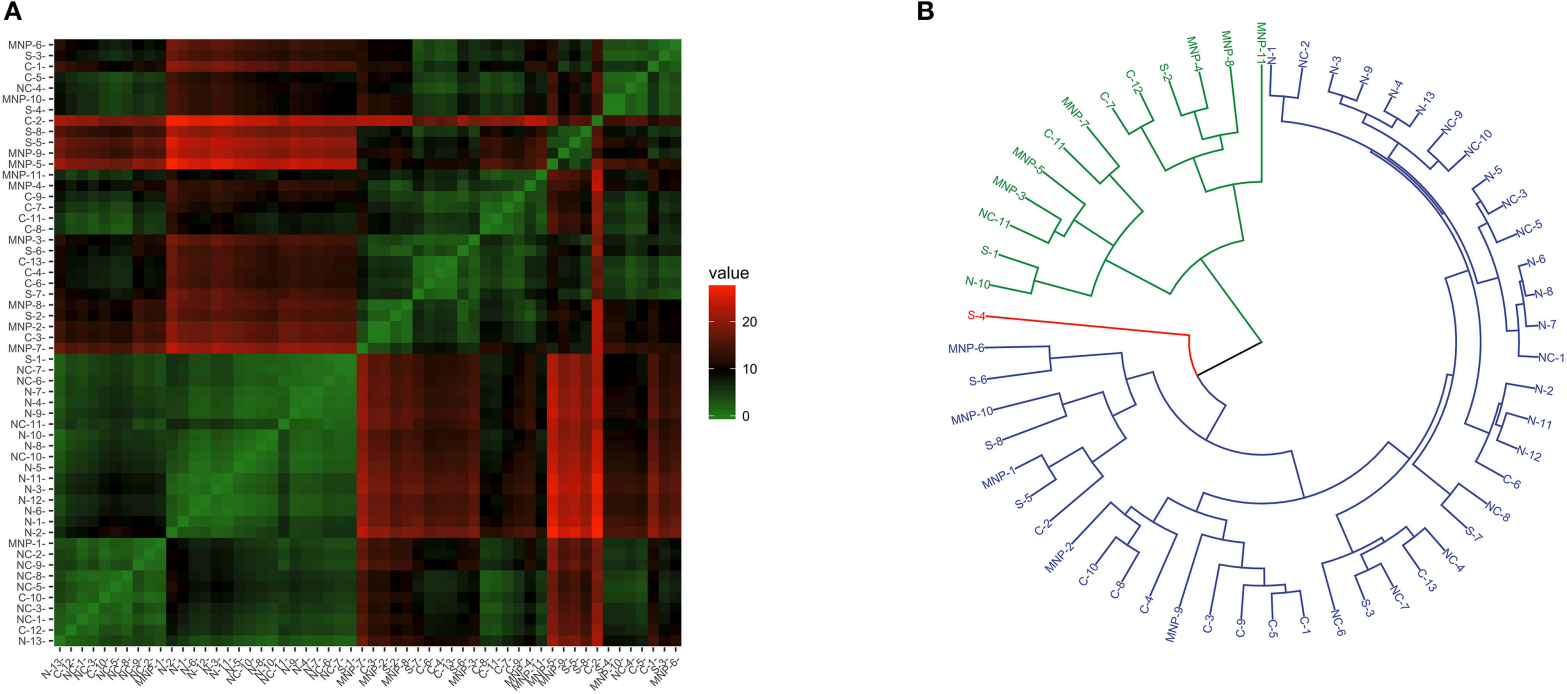
Similarity of NK and CD^8+^ T cell transcription (*Ly49, perforin, Granzyme B, STAT-1, Tbx21*, and *Eomes*) in blood of California sea lions sampled at different regions. **(A)** Heatmap shows similarity clustering of the gene profiles. Green indicates low dissimilarity and red indicates high dissimilarity. **(B)** Dendrogram of gene transcription profile clustering. The tree was built using an Euclidean algorithm. S, South; C, Central; NC, North-Central (Midriff); N, North; MNP, Mexican North Pacific.

When considering transcriptional patterns of all target genes together, no clear grouping emerged (see Figure S5). A few sea lions had a distinct pattern, and each belonged to a different region. The transcriptional profile related to the NK and CD^8+^ cells (considering *Ly49, perforin, granzyme, STAT-1, Tbx21*, and *Eomes* transcription levels) showed some heterogeneity and clustering among sea lions (Figure 3A), and the best dendrogram separated the samples in three main branches (Figure 3B). One of the branches harbored 92% (12/13) of the sea lions sampled in the northern region, and 90.1% (10/11) of the sea lions sampled in the midriff region. Furthermore, sea lions from these regions were grouped into smaller clusters, separating them from samples from the central and southern regions, as well as from the Mexican North Pacific. The second main branch grouped only one sea lion from the northern Gulf of California, two from the midriff region, and a mixture of sea lions from southern and central regions, and from the Mexican North Pacific. The third main branch separated a single sea lion sampled in the southern Gulf of California.

**Figure 4:**
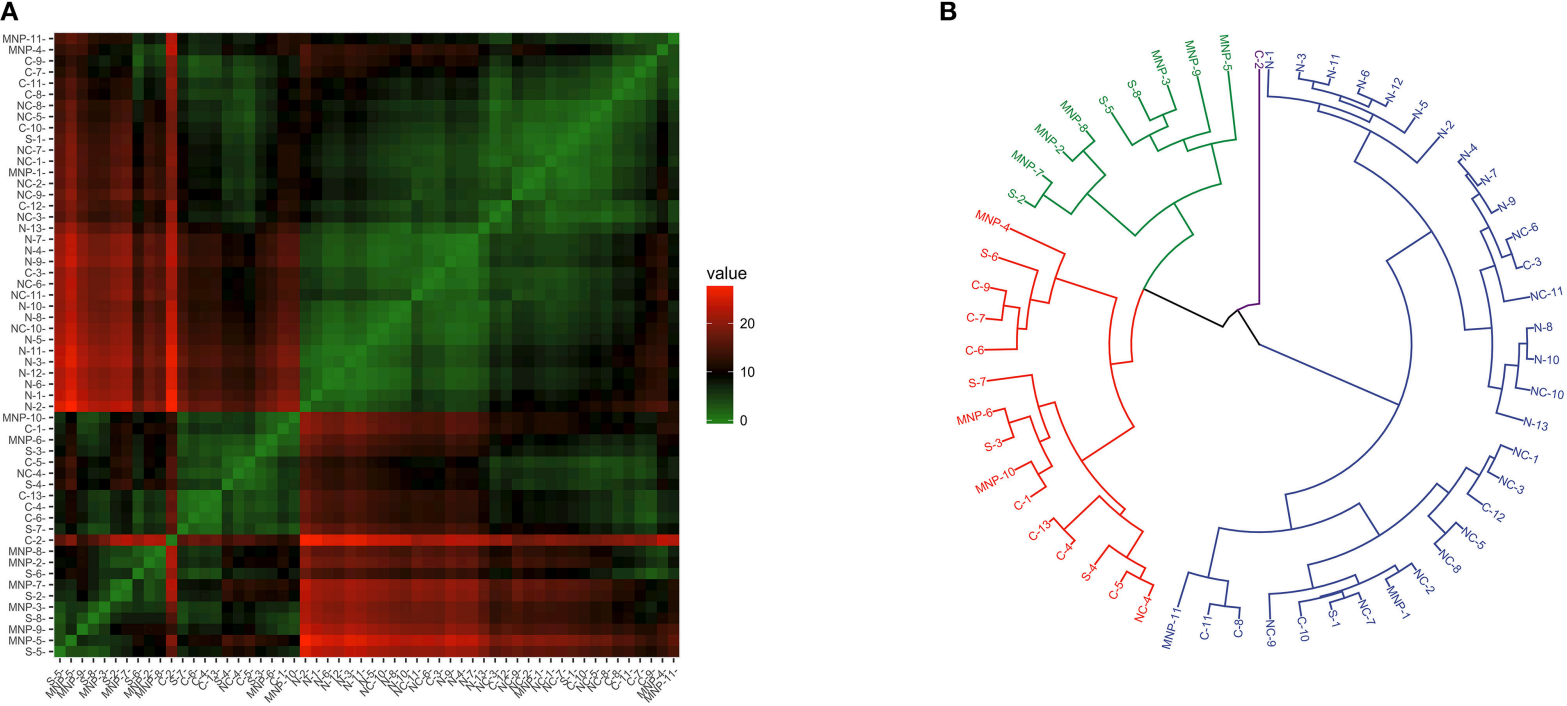
Similarity of Th2 and immunomodulation (*GATA3* and *FoxP3* gene expression) in blood of California sea lions. **(A)** Heatmap shows similarity clustering of the gene profiles. Green indicates low dissimilarity and red indicates high dissimilarity in relative transcription levels of the six genes. **(B)** Dendrogram of gene transcription profile clustering. The tree was built using an Euclidean algorithm. S, South; C, Central; NC, North-Central (Midriff); N, North; MNP, Mexican North Pacific.

The clustering analysis of Th2 and immune modulation profiles revealed some heterogeneity and clustering (Figure 4A), and the dendrogram separated the samples in three main branches (Figure 4B); the first branch grouped all of the sea lions from the northern region, 91% (10/11) of the sea lions from the midriff region and a few sea lions from the other regions; namely, four from the central region, one from the southern region and two the Mexican North Pacific. The second branch harbored sea lions from the southern (7/8) and central (8/11) regions, and from the Mexican North Pacific (8/11). One sea lion from the central region was separated from the rest in its own branch.

**Figure 5:**
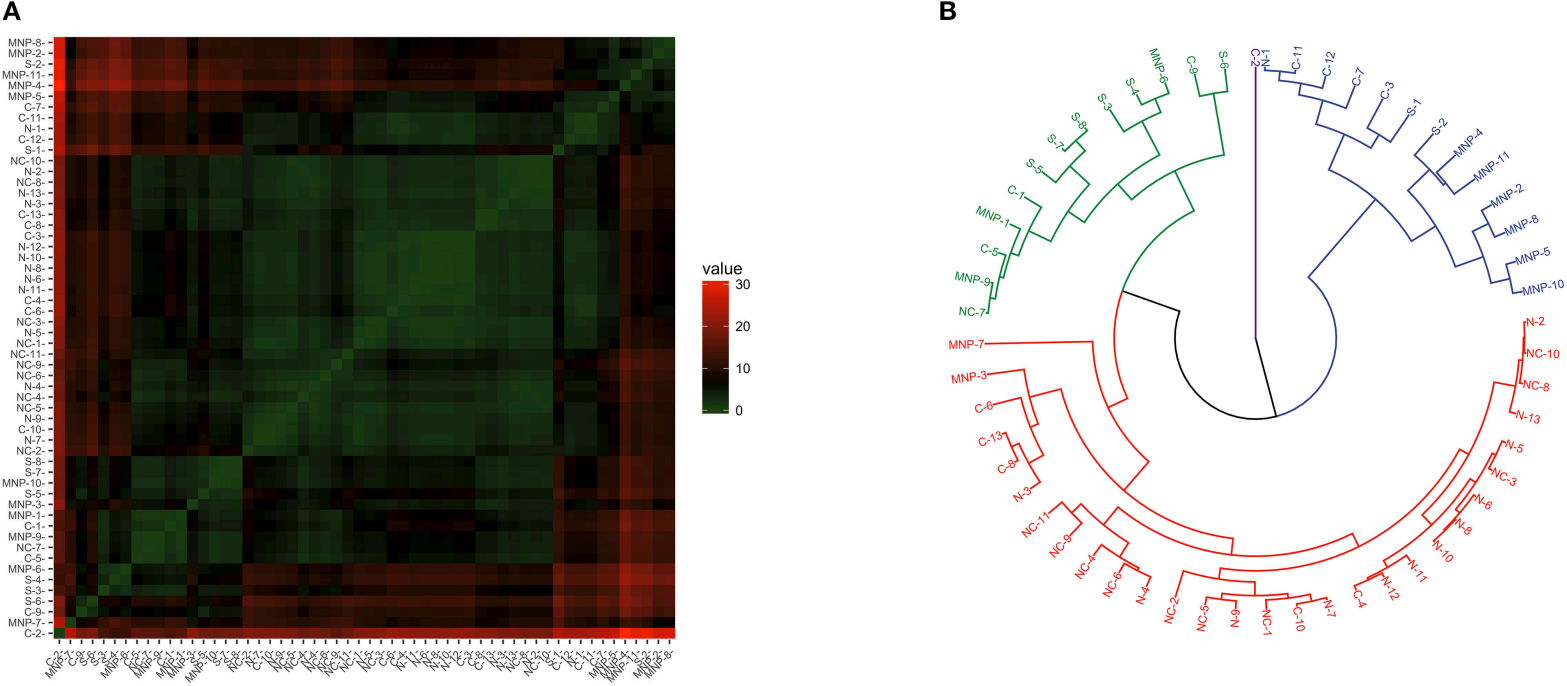
Similarity of Th1/Th2 profile (*GATA3* and *Tbx21* gene expression) in blood of California sea lions. **(A)** Heatmap shows similarity clustering of the gene profiles. Green indicates low dissimilarity and red indicates high dissimilarity in relative transcription levels of the six genes. **(B)** Dendrogram of gene transcription profile clustering. The tree was built using an Euclidean algorithm. S, South; C, Central; NC, North-Central (Midriff); N, North; MNP, Mexican North Pacific.

Clustering patterns of Th1/Th2 profiles were less clear than observed for the other profiles (Figure 5A). The dendrogram separated the samples in four main branches (Figure 5B); one grouping 92% (12/13) of the sea lions sampled in the northern region, 91% (10/11) of the sea lions from the midriff region, 42% (5/12) of the sea lions sampled in the central region, and 18% (2/11) of the sea lions from the Mexican North Pacific. The second and third branches grouped a mixture of sea lions from the central and south regions, and from the Mexican North Pacific. The fourth main branch separated a single sea lion sampled in the central region.

### Gene Transcription and Genital Infection by Oncogenic Viruses

**Figure 6:**
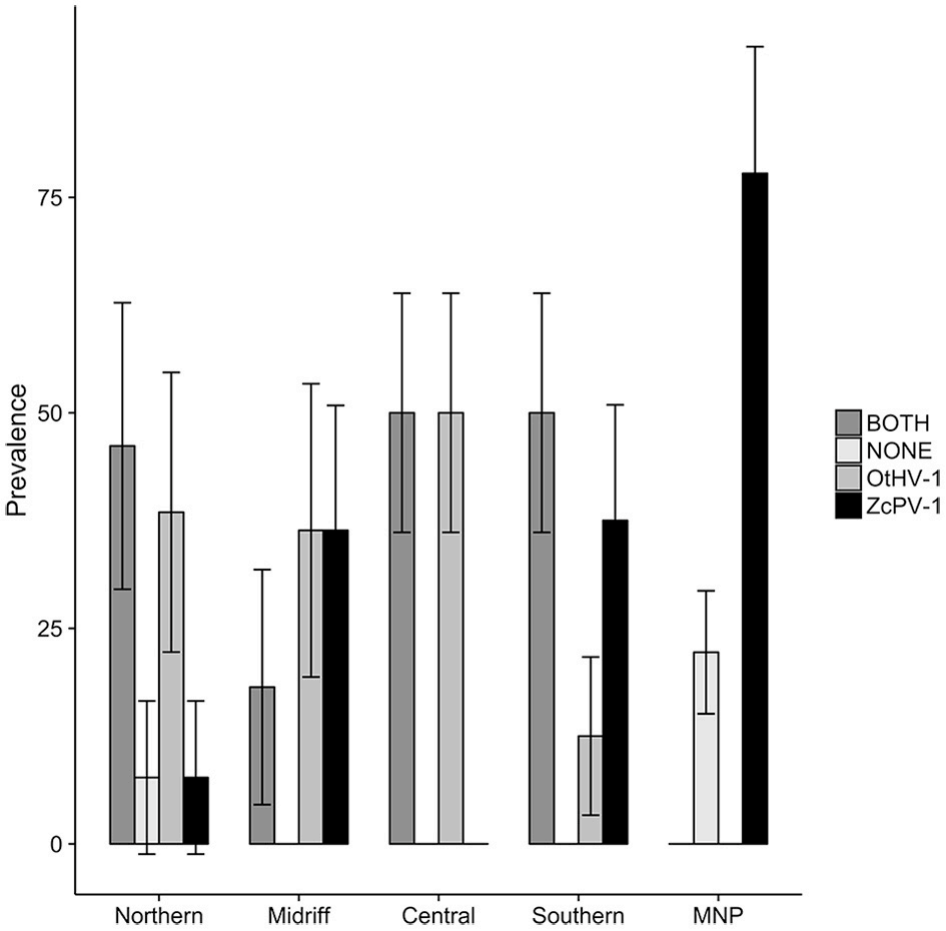
Prevalence of infection by oncogenic viruses in the genital epithelium of California sea lions according to ecological regions within the Gulf of California and the Mexican North Pacific (MNP). ZcPV-1 infections were highest in the Mexican North Pacific followed by the midriff and southern Gulf of California, OtHV-1 infections were highest in the central region, followed by the northern and midriff regions, and concurrent infections by both viruses varied significantly among regions, following the pattern observed for OtHV-1. Bars = ± s.e.

The prevalence of genital viral infections varied significantly across regions (Figure 6). Genital ZcPV-1 infections were highest in the Mexican North Pacific followed by the midriff and southern Gulf of California (Pearson's Chi^2^ = 11.32, *df* = 4, *p* = 0.042), while OtHV-1 infections were highest in the central region, followed by the northern and midriff regions (Pearson's Chi^2^ = 26.39, *df* = 4, *p* = 2.71 × 10^−06^). The prevalence of simultaneous infections by both viruses also varied among regions, with a similar pattern to that observed for OtHV-1 (Chi^2^ = 11.757, *df* = 4, *p* = 0.0231).

**Figure 7:**
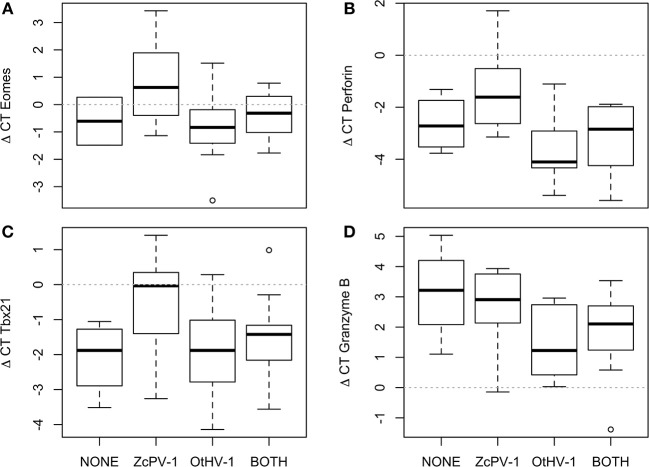
Gene transcription levels varied according to genital infection status. **(A)** *Eomes*, **(B)** *Perforin*, **(C)** *Tbx21*, **(D)** Granzyme. The plot shows the median (thick line), first and third quartile (box), and 95% confidence interval of the median (whiskers).

Infection status impacted transcription of four genes related to cytotoxicity, even when accounting for spatial differences [GLM; *Eomes*: *F*_(3, 29)_ = 4.21, *p* = 0.015, Figure 7A; *perforin*: *F*_(3, 30)_ = 4.743, *p* = 0.008, Figure 7B; *Tbx21*: *F*_(3, 48)_ = 4.16, *p* = 0.012, Figure 7C] granzyme B: *F*_(3, 33)_ = 3.39, *p* = 0.036, Figure 7D. The pattern was consistent and contrasting between viruses. Namely, sea lions infected by OtHV-1 had transcription levels similar to or lower than those of non-infected sea lions and sea lions simultaneously infected by both viruses. In contrast, sea lions infected by ZcPV-1 had significantly higher transcription levels. None of the other target genes were significantly influenced by genital infection status. In order to examine whether the expression levels of *Tbx21* and *Eomes* in sea lions with single infections (either ZcPV-1 or OtHV-1) or with concomitant infections were associated with the up-regulation of inhibitory receptors or suggested a skewed maturation phenotype, we examined the relationship of transcription levels of these genes with those of the inhibitory receptor *Ly49*. We found no correlation for sea lions infected simultaneously by both viruses (*r*^2^ = 0.04, *p* = 0.52), nor was there a significant relationship for sea lions that were infected only by OtHV-1 (*r*^2^ = 9.11, *p* = 0.43) or only by papillomavirus (*r*^2^ = 0.12, *p* = 0.26; see Figure S6).

### Gene Transcription and Inflammation

**Figure 8:**
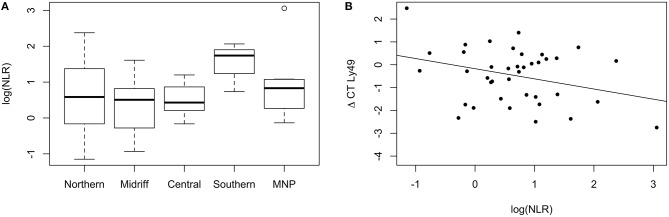
**(A)** Neutrophil to Lymphocyte Ratio (NLR) of California sea lions from different regions. The variance of NLR was larger in sea lions from the northern and midriff regions. The plot shows the median (thick line), first and third quartile (box), and 95% confidence interval of the median (whiskers). MNP: Mexican North Pacific. **(B)** Relative transcription levels of Ly49 are explained by the NLR of California sea lions. Higher NLR values were associated with lower levels of transcription (Adjusted *R*^2^ = 0.078).

The marker of chronic systemic inflammation and stress here used, NLR, did not vary significantly across regions. However, the variance differed significantly [*F*-test; *F*_(39, 55)_ = 0.37, *p* = 0.002], being larger for sea lions from the northern and midriff regions (Figure 8A). Eight percent of the variation in transcription levels of *Ly49* was influenced by the NLR, and higher NLR values were associated with lower levels of transcription [*F*_(1, 38)_ = 4.19, *p* = 0.048, Figure 8B]. None of the other target genes were significantly impacted by the NLR of the sea lions sampled.

## Discussion

Our study examined the transcriptional levels of key genes related to cytotoxic responses to viruses and tumor cells, as well as those that are involved with immune modulation in free ranging California sea lions. We found indication of regional differences in the expression of genes related to Th1 responses and immune modulation, and detected different transcription levels in sea lions infected by genital papillomavirus compared to those infected by genital gammaherpesvirus. Furthermore, when analyzing the gene expression profiles according to functional groupings, a north to south division became apparent, where sea lions from the northern and midriff regions were clustered together, while most sea lions from the central and southern regions, as well as from the Mexican North Pacific, clustered together in another group. As is unavoidably the case when studying free-ranging species, our results are correlative, and impede the inference of direct causation. Furthermore, given that we focused on gene transcription rather than on protein expression, we cannot make inferences regarding the expression of the gene products themselves. Nonetheless, the patterns observed were consistent with what is known for herpesvirus and papillomavirus infections in humans and model species, and the relationships in gene expression levels between the markers selected are strongly suggestive of differential ecological impacts on immune effectors. Owing to the paucity of information regarding pinniped immunity, we will discuss our findings in light of what is known for humans and model animals.

Transcription of two genes, *granzyme B* and *perforin*, directly involved with cytotoxic responses did not vary amongst ecological regions. *Granzyme B* and *perforin* are expressed by NK and CD^8+^ T cells, and are crucial to induce apoptosis of transformed or virus-infected cells (44). Both genes are induced progressively upon activation by antigen-presenting cells. *Granzyme B* encodes a serine protease (granzyme B) that is released together with perforin, a glycoprotein encoded by the *perforin* gene. Perforin forms a pore in the membrane of the target cell, allowing granzyme B to enter the target cell and trigger apoptosis via the activation of various caspases, generation of mitochondrial reactive oxygen species, and DNA fragmentation (45). The nearly 5-fold difference in transcription levels between both genes implies that individual sea lion NK and CD^8+^ T cells transcribe different combinations of *perforin* and *granzyme B*, and that each gene is differentially regulated at the single cell level (46). It is possible that, as has been reported for humans (47), granzyme B could exhibit some perforin-independent activity in the California sea lion, Alternatively, it is also plausible that the results indicate that for every transcribed mRNA of *perforin*, more *granzyme B* transcription is required.

We also selected *Ly49* as a marker of NK function, and found its expression to be slightly downregulated with respect to the reference genes in nearly all of the sea lions. *Ly49* encodes functional inhibitory NK cell transmembrane receptors in the California sea lion (48). Inhibitory NK receptors are essential for detecting missing or altered MHC class I molecules in target cells (49). The low levels of expression of *Ly49* could reflect that the adult sea lions have a small population of peripheral mature NK cells that are undergoing *Ly49* gene activation. If so, most of the *granzyme B* and *perforin* would have been transcribed by peripheral CD^8+^ cells, as they tend to be more abundant than NK cells in blood (50). This possibility is strengthened by the loose correlation observed between the transcription levels of *Ly49* and those of *granzyme B* and *perforin*. Future studies should aim to explore the functional role of other NK cell transmembrane receptors, such as the killer cell lectin like receptor 1 gene (*nkg2d*), whose role for modulating cytotoxicity has been associated with susceptibility to papillomavirus-related cancers in humans (51).

Relative expression levels of *Eomes, Tbx21*, and *STAT-1*, genes also expressed by NK and CD^8+^ T cells (52), was closely correlated, as would be expected in a Th1 (pro-inflammatory) environment (53, 54). Furthermore, these genes exhibited regional variation in transcription. *Eomes* was downregulated in most sea lions from the midriff and central regions, but upregulated in sea lions from the northern and southern regions, as well as in the Mexican North Pacific, while *Tbx21* and *STAT-1* transcription levels showed a clear north-to-south increase within the Gulf of California and reached highest levels in the Mexican North Pacific. When considering the transcriptional profile of NK and CD^8+^ cells, the hierarchal clustering analysis revealed two main profiles. Sea lions from the northern and midriff regions exhibited one of them, while most sea lions from the southern region and the Mexican North Pacific exhibited the other. Sea lions from the central region exhibited one or the other profile, and a few sea lions from the southern region and the Mexican North Pacific exhibited a more “northern” transcriptional profile. This suggests a spatial difference in the prevalence of intracellular pathogens or differential exposure to other factors that could cause cellular insults.

The encoded products of *Eomes, Tbx21*, and *STAT-1* (Eomesodermin, T-bet, and STAT-1, respectively) are essential to Th1 cell differentiation, and are consequently important for downstream anti-viral and anti-tumoral responses (16). *Eomes* gene expression can be upregulated by type I interferon signaling in CD^8+^ T cells (55), while *Tbx21* is upregulated in response to stimulation by antigens and macrophage-derived cytokines (56). STAT-1 regulates *Tbx21* transcription, and in turn, high levels of *Tbx21* influence the transcription of *perforin* and *granzyme B* in antigen-stimulated cells (57). Furthermore, T-bet promotes and sustains virus-specific CD^8+^ responses, particularly during chronic infections (58). The relatively higher transcription levels observed in sea lions from the southern Gulf of California and Northern Pacific could indicate increased exposure to intracellular pathogens or anti-tumoral activity. Two results add support for this proposed explanation. First, sea lions with genital infections by papillomavirus had increased expression of *Eomes, Tbx21*, and *perforin*, and second, papillomavirus infections were significantly higher in the southern Gulf of California and Mexican North Pacific. Research on human papillomavirus has shown that the oncogenic proteins E6 and E7 can induce T-cell responses in individuals with a functional immune system. The responses, characterized by higher densities of T cells that express eomesodermin (16), T-bet (17), and perforin, among other markers help clear, or avoid, the occurrence of cervical neoplasia (17). Based on our results, we suggest that ZcPV-1 induces systemic immune responses in otherwise healthy California sea lions.

An opposite pattern was observed for OtHV-1. Here, genetic expression of *Eomes, Tbx21*, and *perforin* was lower in infected sea lions. In marked contrast to papillomavirus, whose tropism is restricted to the basal layer of the epithelium (59), gammaherpesviruses have a wider tropism that includes epithelial cells and B-lymphocytes (60). In humans, active gammaherpesvirus infections are most often associated with severe immunosuppression, and the viruses can switch to latency, establishing lifelong persistent infections, which can then lead to lymphomas and carcinomas, as well as lymphoproliferative disorders of NK and T cells (61). Immune control of gammaherpesviruses infections is complicated, as they have evolved various immune evasion strategies (62). Unfortunately, knowledge about OtHV-1 pathogenesis is currently limited, but based on our results, it is tempting to speculate that detectable infections in the genital tract will be much more common in sea lions with a suboptimal immune status, and that infections will, in turn, avoid cytotoxic immune responses, further increasing the risk of malignant transformation of infected cells. This hypothesis is strengthened by two findings: one, a relative decrease in lymphocyte counts was observed in herpesvirus-infected sea lions compared to sea lions with papillomavirus or those with no apparent infections (see Figure S7); and two, when comparing levels of transcription of genes related to cytotoxicity among apparently uninfected sea lions, sea lions infected by OtHV-1, ZcPV-1, and by both viruses simulaneously, levels of *Eomes, perforin*, and *tbx21* of sea lions infected by OtHV-1 were equal to or lower than uninfected sea lions. Interestingly, simultaneous infection of the genital epithelium by OtHV-1 and ZcPV-1 resulted in a pattern of transcription similar to that of OtHV-1 alone. This was unexpected, and implies that co-infections might be an important risk factor for transformation of the genital epithelium, owing to a reduction in anti-viral and anti-tumor activity. A recent study reported coinfection by herpesviruses and papillomaviruses in genital tumors of Atlantic bottlenose dolphins, *Tursiops truncatus* (63) and it has already been shown that co-infections by oncogenic papillomaviruses and herpesviruses increase the risk of cervical carcinoma in humans (64). Based on our results, we believe that the potential role of papillomavirus as a co-factor in for sea lion urogenital carcinoma should be reconsidered [see (3)].

Transcription levels of the two genes selected as markers of Th2 responses and immune modulation varied among regions, once again with a north to south gradient. *GATA3* was downregulated in sea lions from the northern and midriff Gulf of California, and upregulated in sea lions from the central and southern Gulf of California and Mexican North Pacific, where levels were the highest. *FoxP3* mirrored the spatial pattern, although transcription levels of this gene were, on average 4.5-fold lower than that of *GATA3*. Both genes were correlated, as would be expected since *GATA3* modulates *FoxP3* expression and activity (65), however, *GATA3* only explained 13% of the variation in *FoxP3* levels, and a number of individuals deviated greatly from the predicted regression. *GATA3* is a transcription factor expressed by mature CD^8+^ T cells, and it plays a key role in leading to the differentiation of Th2 cells, regulating T cell development, proliferation, metabolism, and maintenance. In particular, it promotes development of anti-inflammatory Th2 and Th9 cells, and suppresses pro-inflammatory Th1 and Th17 differentiation as well as B cell development (65). In turn, *FoxP3* is highly expressed by CD^4+^ T regulatory (Treg) cells, and it is essential to ensure immune homeostasis by suppressing (or destroying) active leukocytes via stimulating direct cytotoxicity, depleting growth factors in the extracellular environment, and secreting anti-inflammatory cytokines and co-inhibitory molecules that can modulate the activity of antigen-presenting cells (65). Expression of *FoxP3* is particularly important to control excessive anti-viral responses and limit the extent of immunopathology (66). Based on the fact that (i) several of the genes involved with promoting pro-inflammatory and cytotoxic responses were upregulated in sea lions from the central and southern Gulf of California and Mexican North Pacific, and (ii) these are the regions where transcription of *GATA3* was increased, and that expression of *GATA3* is known to be tightly regulated by TCR and pro-inflammatory cytokine stimulation, which in turn responds to active CD^8+^ and NK activity (67), it is possible that sea lions in these regions are experiencing active responses to one or more viruses, and are, at the same time, experiencing some level of immunomodulation to avoid damage associated with these responses. The hierarchal analysis showed some support for this explanation, as the dendrogram revealed two large groups that separated sea lions from the northern and midriff region from those from the central and southern Gulf of California, and the Mexican North Pacific. Thus, it is likely that differences in viral exposure, as well as other as yet unidentified environmental and ecological variables are impacting sea lion immune modulation in addition to their cytotoxic immune activity. Having found that the NLR was higher in sea lions from the northern and midriff region implies that these individuals are undergoing chronic inflammation. However, as our study focused exclusively on assessing gene transcription levels, rather than proteins, this possibility will need to be examined in more detail in the future by quantifying cytokine levels in the blood.

The ratio of *GATA3* to *Tbx21* (here Ga/Tb) is an indirect measure of the Th1/Th2 profile of an individual (39). Both transcription factors cross-regulate each other, as T-bet modulates GATA-3 function and Th2 cytokines block Th1 differentiation (66). The interrelationship of these molecules and the effector responses that they regulate define the host response and, therefore, influence the outcome of a given infection. While mean Ga/Tb did not vary among regions, when analyzing the Th1/Th2 profile by hierarchal clustering, a north to south pattern emerged once again, with one group comprised by sea lions from the midriff, northern, and one comprised by sea lions from the southern region. As observed for transcriptional markers of cytotoxicity, sea lions from the central region exhibited either one the responses.

The observed patterns in the transcriptional profiles of genes related to cytotoxicity, Th2 and immunomodulation, and Th1/Th2 balance were congruent. In all cases there appeared to be a distinction between patterns exhibited by sea lions from the northern and midriff regions and those exhibited from the southern region and the Mexican North Pacific. While differences in transcription levels do not necessarily indicate differences in the synthesis of the proteins of interest due to transcriptional and post-transcriptional regulation (68), the congruence in the transcriptional profiles observed cannot be ignored. In particular, these profiles appear to be related to genital infection by two oncogenic viruses, and are likely to reflect other ecological and environmental factors, such as differences in contaminant levels (7, 20, 21) and availability of energetic resources (25) to implement immune responses (32). These possibilities will need to be addressed in future studies. Given the role of NK and CD8+ cells for tumor surveillance and anti-viral responses (15–17), the significance of OtHV-1 in the development of sea lion urogenital carcinoma (2, 3), the potential importance of ZcPV-1 in oncogenesis (63), and the stark difference in the prevalence of urogenital carcinoma between sea lions from the US and Mexican waters (18), the patterns observed are likely to be biologically significant.

Marine mammals, as apex predators, are continuously exposed to xenobiotics that can impact their immune competence. This is particularly problematic, given the growing number of emerging pathogens in the marine environment and the rise in disease conditions and unusual mortality events in the past decades. In this context, it is relevant and timely to increase our understanding of the factors that can hinder different immune effectors of these species (69), particularly of those that are prone to develop chronic and deadly diseases, such as urogenital carcinoma, which could be considered sentinels of immune competence (70). Our study is a first approach to profile the transcriptional patterns of key immune effectors of free-ranging California sea lions, and their association with ecological regions and infection by oncogenic viruses. The observed results and suggested patterns add insight to our understanding of immune competence of marine mammals, and may help elucidate the marked difference in the number of cases of urogenital carcinoma in sea lions from US waters and other areas of their distribution.

## Data Availability

The datasets generated for this study are available on request to the corresponding author.

## Author Contributions

KA-W conceived, designed and coordinated the study. IP conducted gene expression assays, statistical analyses, and drafted the manuscript. AF-M conducted hematological analysis; MF-C and RÁ-M assisted with statistical design, analysis and interpretation of data. FG-dlR and LS-G performed pathogen detection assays. All authors read and commented the final draft of the manuscript and gave final approval for publication.

### Conflict of Interest Statement

The authors declare that the research was conducted in the absence of any commercial or financial relationships that could be construed as a potential conflict of interest.
