# Supplementary material for: Transcriptional Profiles of California Sea Lion Peripheral NK and CD+8 T Cells Reflect Ecological Regionalization and Infection by Oncogenic Viruses
Source: Front Immunol. 2019 Mar 12;10:413. doi: 10.3389/fimmu.2019.00413 (PMC6422979; doi:10.3389/fimmu.2019.00413)
Supplement: Supplementary file 1 [file Data_Sheet_1.PDF]

Supplementary material

Assessment of RNA integrity

RNA integrity was assessed by electrophoreseis in a 1% agarose gel stained with ethidium bromide and purity was determined by Nanodrop spectrophotometry (Nanodrop 2000, Thermo Fisher Scientific, USA). Good quality samples were those that had no evidence of RNA degradation, showed clear 28S and 18S rRNA bands, as can be seen below (Fig. S1), and whose A_{260}/A_{280} ratio was between 1.75 and 2.10. Five of the RNA samples did not clearly show the two rRNA bands despite having a good A_{260}/A_{280} ratio. In these cases, we re-extracted the RNA and reassessed the quality. In all of these cases, we were able to confirm that quality was adequate to proceed with reverse transcription.

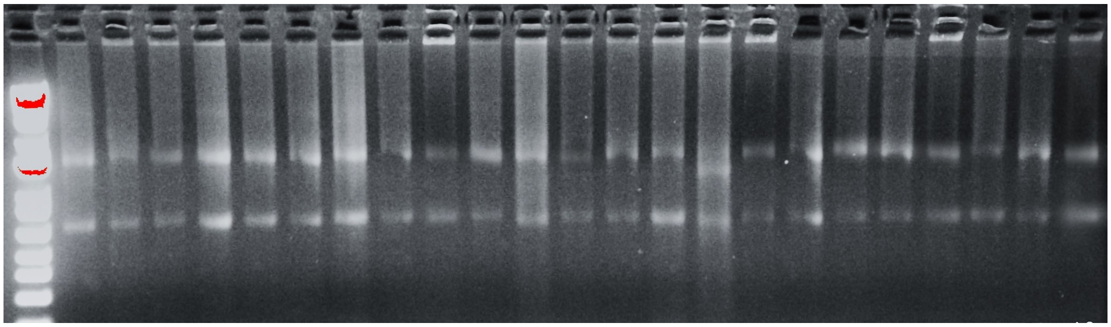

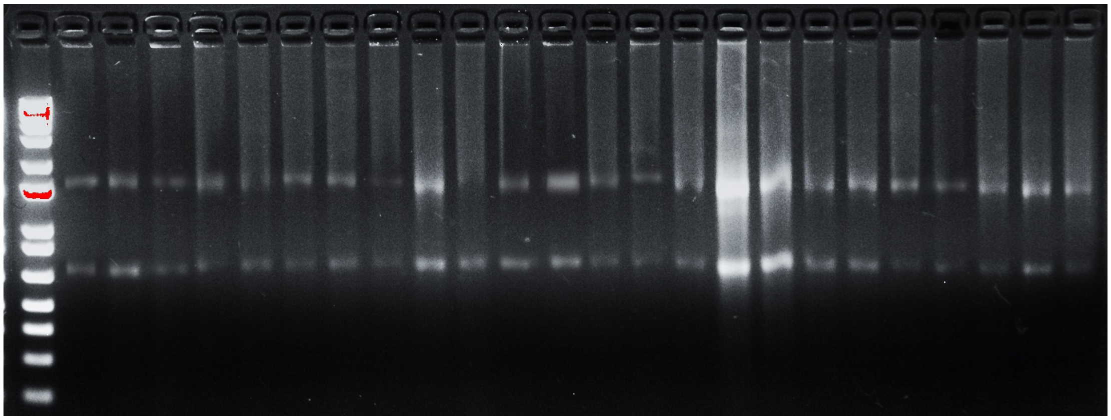

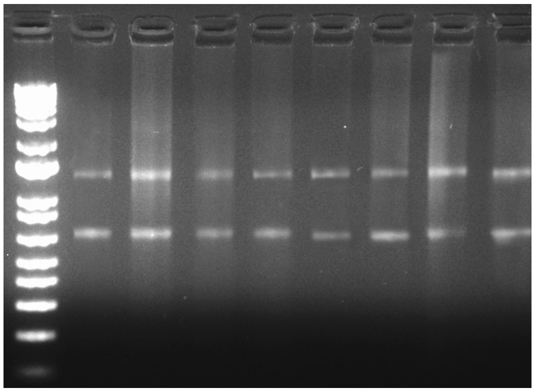

Fig. S1. Extracted RNA electrophoresed on a 1% agarose-gel stained with ethidium bromide. RNA was extracted from the buffy coat of free-ranging adult California sea lions. Top panel) RNA from sea lions from the northern region (lanes 2-16) and the northcentral region (lanes 17-24), Middle panel) RNA from sea lions from the southern region (lanes 2-8), the central region (lanes 9-16), and the northcentral region (lanes 17-25). Bottom panel) RNA from sea lions from the Mexican North Pacific. In all cases, 2 µL of RNA (100 µg/ µL) of RNA were loaded. 5 µL of Hyperladder 1 Kb (Bioline, cat. 33053) is shown in lane 1 of all three gels.

Supplementary material

Reference gene stability

We selected two genes as reference genes (ribosomal protein S5, *RPS5*; and hypoxanthine phosphoribosyltransferase 1, *HpRT*) for the qRT-PCR assays. These were selected because they are expressed in all nucleated cells, and had previously been found to be stable in circulating leukocyte transcripts from free-ranging adult California sea lions and to outperform another reference gene tested, glyceraldehyde 3-phosphate dehydrogenase *GAPDH* (Vera-Massieu 2015). In order to comply with the Minimum Information for Publication of Quantitative Real-Time PCR Experiments (MIQE) guidelines, here, we present the steps taken to determine the that *RPS5* and *HpRT* were suitable as reference genes.

Due to the lack of genomic resources for the study species, for each of the three genes of interest we designed primer pairs within highly conserved regions of sequences reported for related species that were available in GenBank (otariid and phocid species when available, and the domestic dog, *Canis familiaris*). The primer sequences used were: *GAPDH*, F: 5'- TGTCCCCACCCCCAATGTATC, R: 5'-CTCCGATGCCTGCTTCACTACCT; *RPS5*, F: 5'- TCACTGGTGAGAACCCCCT, R: 5'-CCTGATTCACACGGCGTAG; and *HpRT*, F: 5'- AGCTTGCTGGTGAAAAGGA, R: 5'-TTATAGTCAAGGGCATATC. Expected amplicon size varied between 122 and 96 bp.

We determined the efficiency and specificity of each of the three primer sets. We first amplified each primer pair by PCR and used the products as a template for the construction of the standard curves for both reference genes. For this, three amplified products of each gene were run on a 1.9% agarose gel and the excised bands were cleaned using the QIAquick gel extraction kit (Qiagen, UK). PCR quantity was measured with a spectrophotometer (Nanodrop 2000, Thermo Fisher Scientific, USA) and dilutions were made to obtain stocks containing 10^2 to 10^8 copies of PCR product per µl. Each dilution was run in triplicate in a CFX Connect™ Real-Time PCR Detection System (BioRad, USA) as follows: 95ºC for fifteen minutes, followed by 40 cycles of 15 seconds at 95ºC, one minute at 55ºC (during which the plate was read) and 72ºC for 1 minute. The ending cycle was kept at 95ºC for 15 seconds and a final step for the melting curve at 60 to 90ºC (0.5ºC increase and 15 seconds of wave length measurement for each temperature). Reaction specificity was monitored by melting curve analysis, using a final data acquisition phase of 60 cycles of 65ºC for 30 seconds. A single peak was observed for each of the gene products (Fig. S2).

The logarithm of the copies of PCR product obtained for each threshold value (Ct) was plotted against the Ct values to calculate the linear correlation coefficient (R^2) for each primer pair. The qPCR amplification efficiencies (E) were calculated based on the slope of the curve ($E=10^{1/-slope}- 1$) for each set of primers (Schmittgen & LIvak 2008). Amplification of each primer set produced a single and well-defined band and had a unique dissociation curve. The R^2 of the primer pairs ranged between 95% and 109.7% and the E ranged between 0.951 and 0.984 (Fig. S3).

Supplementary material

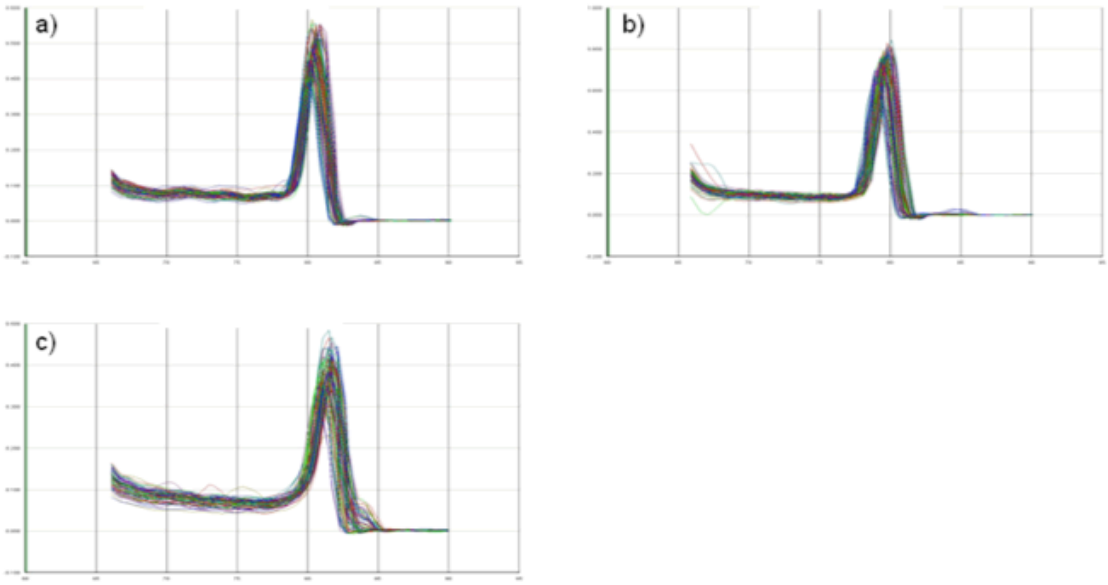

Figure S2. *qRT-PCR dissociation curves of the three reference genes examined. a) HpRT*, b) *RPS5*, and c) *GAPDH. Melting curve analyses images were obtained with a CFX Connect™ Real-Time PCR Detection System (BioRad, USA).*

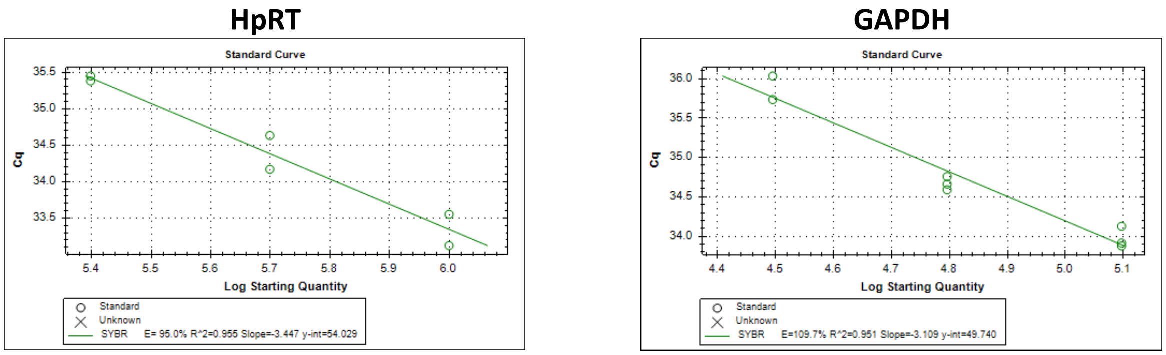

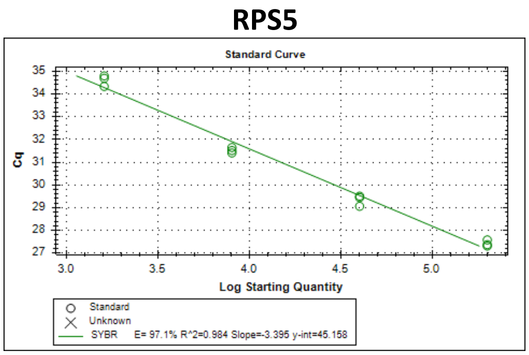

Figure S3. Efficiency and correlation coefficients of the primer pairs used to amplify the reference genes *HpRT*, *RPS5*, and *GAPDH*.

Supplementary material

Gene expression values were analyzed using the freeware packages BestKeeper[1] (Pfaffl et al. 2004), geNorm[2] (Vandesompele et al. 2002) and NormFinder[3] (Schmittgen & Livak 2008). According to Bestkeeper, the most most stable gene was *RPS5* which had the highest correlation coefficient (0.96). Sample integrity was of high quality and all intrinsic variances (InVar [±x-fold]) ranged between 0.06 and 0.94. GeNorm showed that the expression of the three genes was stable, and the highest M value detected (GAPDH: 0.98) was lower than the default limit (M=1.5). The two most stable genes were *RPS5* and *HpRT* (Fig. S4). The optimal number of control genes needed for qPCR normalization in our study system was two genes. Finally, according to NormFinder, the gene with the best stability value was RPL5 (stability value: 0.18; Fig. S4) and the most suitable gene combination was *RPS5-HpRT*, having a joint stability value of 0.19. *RPS5* and *HpRT* were considered the best reference genes to evaluate gene expression by means of relative quantitation of transcription levels.

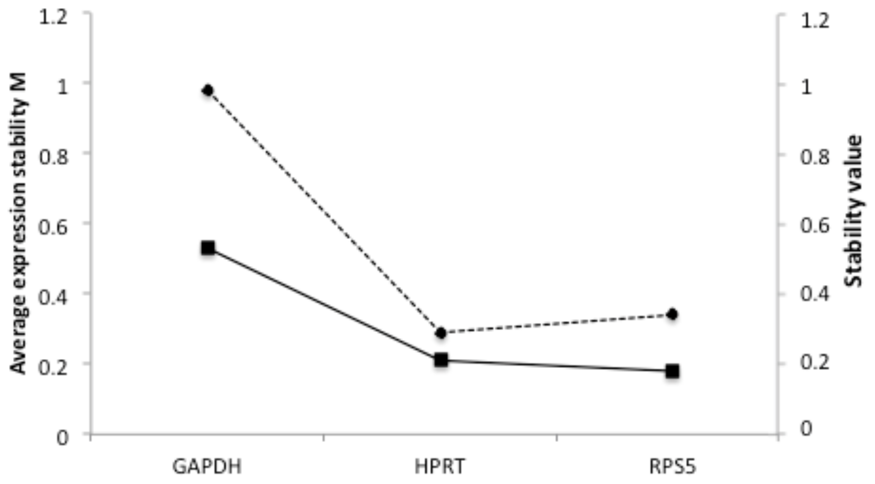

Figure S4. Stability of the reference gene candidates. Average expression stability (M) values were calculated with geNorm (left axis; M value from the least stable on the left to the most stable on the right: 0.981, 0.243 and 0.341; dotted line). Stability values were calculated with NormFinder (right axis; S value from left, least stable, to right, most stable: 0.532, 0.213, 0.182; black line).

qRT-PCR standardization and primer efficiency

RPS5 and *HpRT* performed adequately at the annealing temperature ranges for the target genes; namely, the efficiencies of the target and reference genes were within the accepted 10% range of each other, which made it possible to use the Delta Ct method (Schmittgen & Livak 2008). The efficiency of the primers ranged between 90.6 and 112.8%, and the coefficient of determination (R^2) were >0.949 (Fig. S5).

[1] https://www.gene-quantification.de/bestkeeper.ht
[2] https://genorm.cmgg.be/ (Used gNormv3.xls version with Excel 2007)
[3] https://www.moma.dk/normfinder-software

Supplementary material

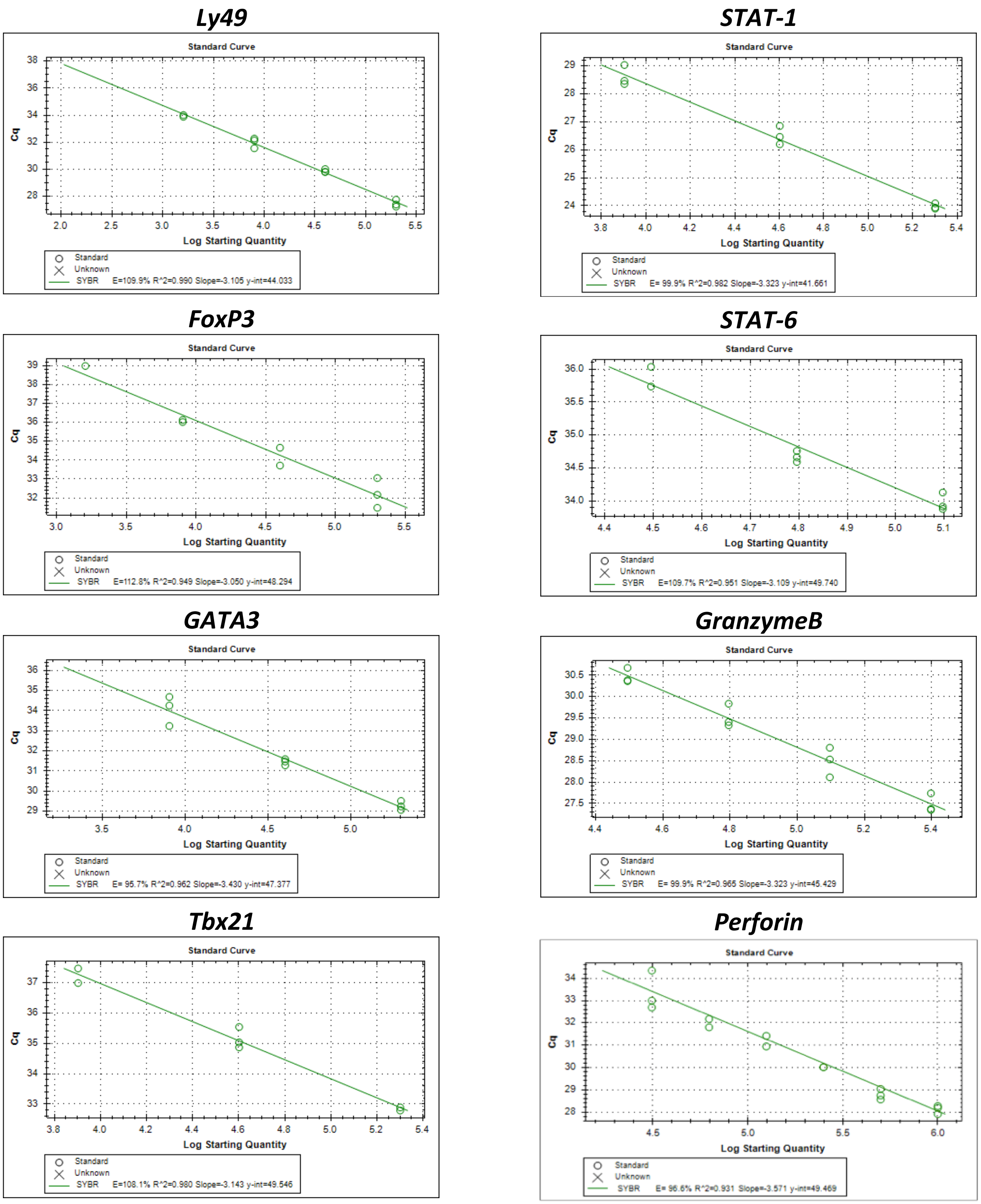

Figure S5. Efficiency and correlation coefficients of the primer pairs used to amplify the reference genes HpRT, RPS5, and GAPDH.

Supplementary material

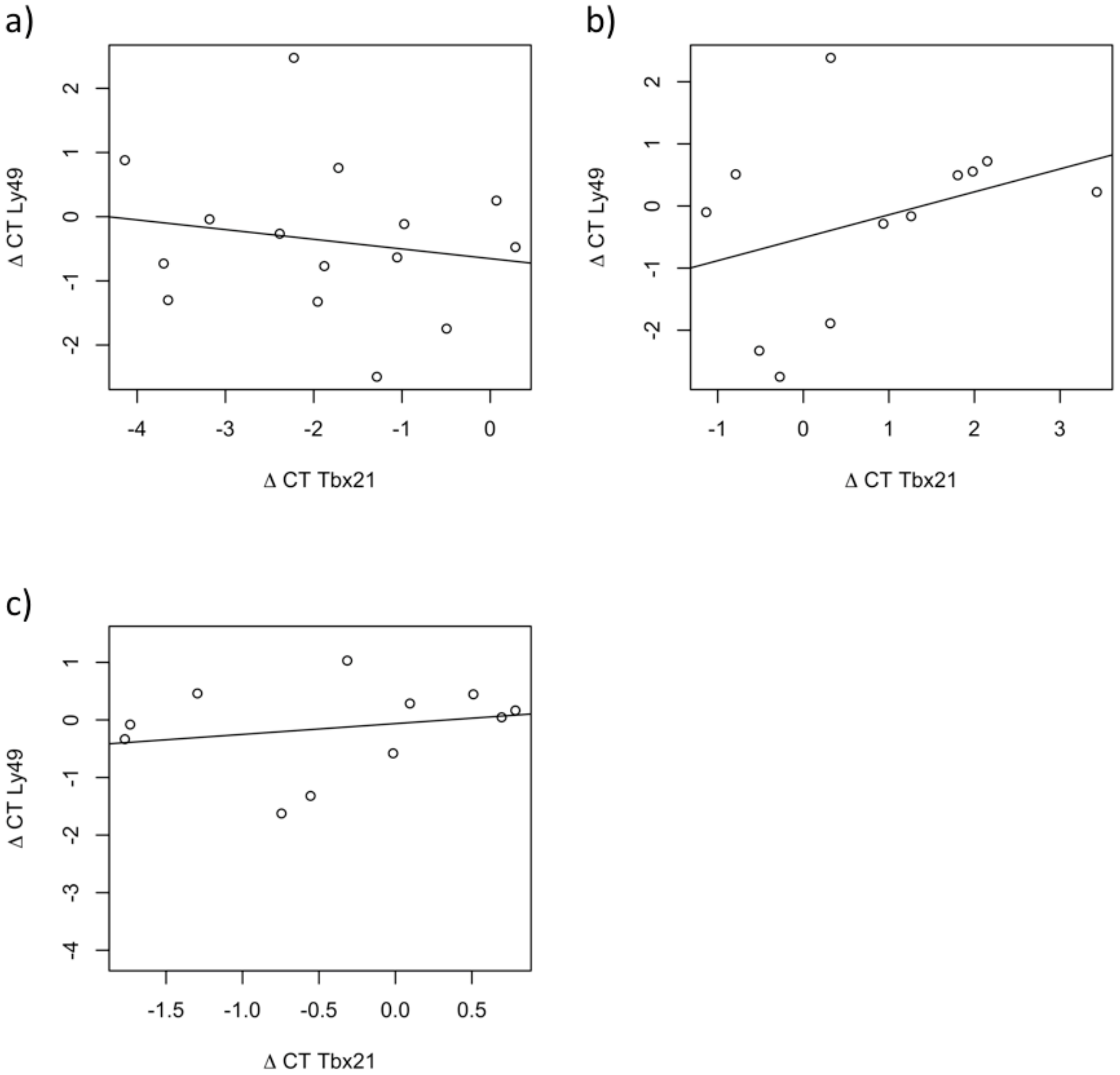

Figure S6. Relationship between the transcription levels related to Th1 differentiation (*Tbx21*) and transcription levels of *Ly49* inhibitory receptor in adult California sea lions according to infection status a) OtHV-1 infection, b) ZcPV-1 infection, c) concomitant infection. The lack of a significant relationship suggests viral specificity of the responses.

Supplementary material

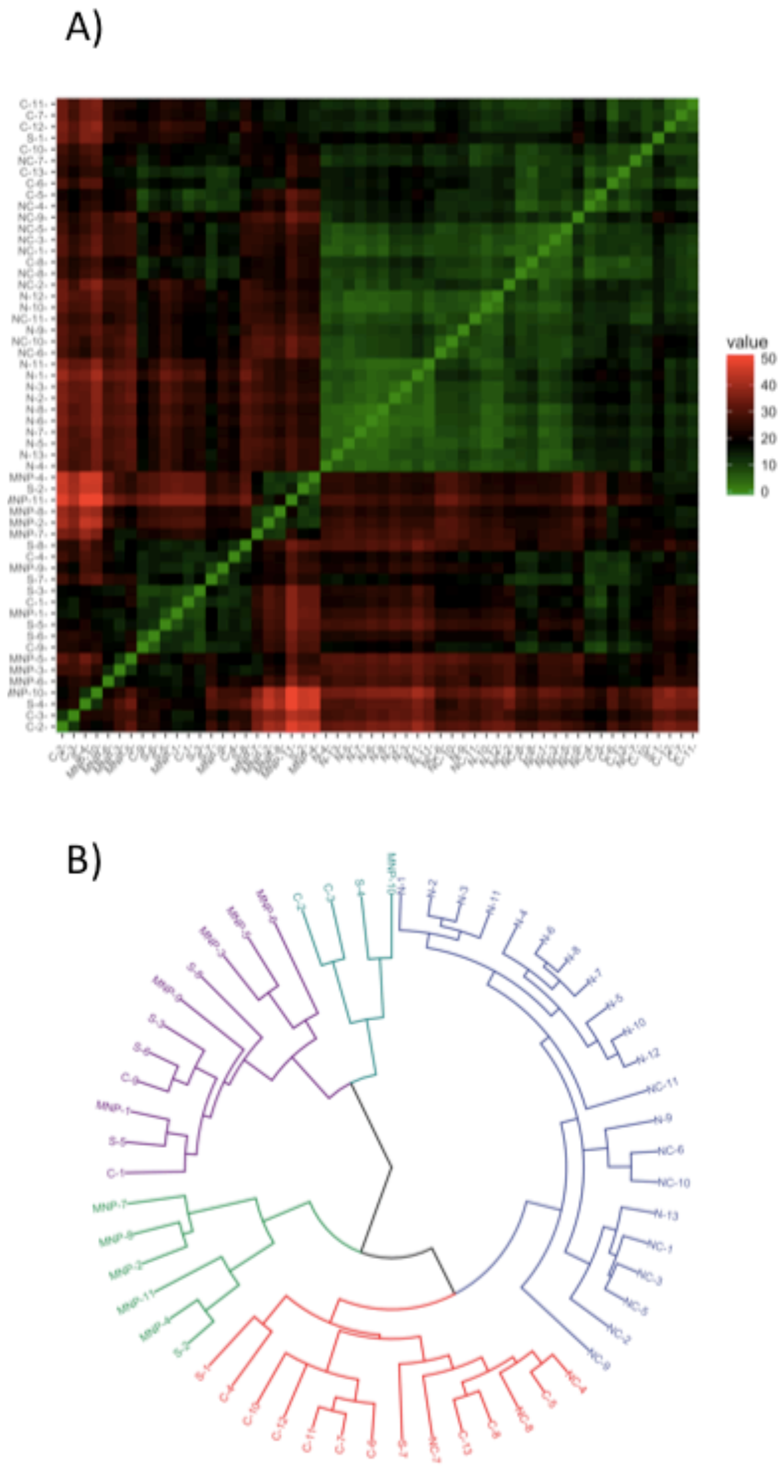

Fig. S6. Similarity of transcription levels in the blood of adult California sea lions from different regions, taking into account all genes included in this study (*Ly49*, *perforin*, *granzyme*, *STAT-1*, *Tbx21*, *Eomes*, *GATA3*, and *FoxP3*). A) Heatmap shows similarity clustering of the gene profiles. Green indicates low dissimilarity and red indicates high dissimilarity. B) Dendrogram of gene transcription profile clustering. The tree was built using an Euclidean algorithm. S: South, C: Central, NC: North-Central (Midriff), N: North, MNP: Mexican North Pacific.

Supplementary material

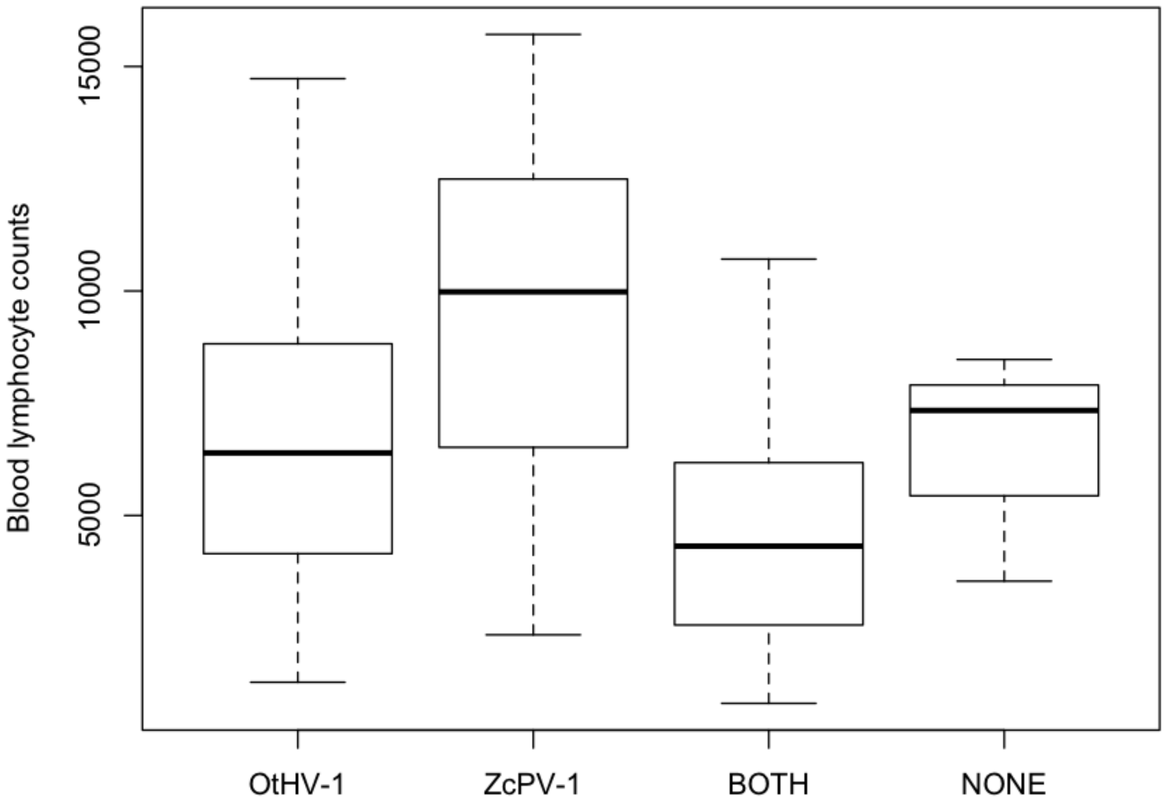

Fig. S7. Relative decrease in lymphocyte blood counts in herpesvirus-infected sea lions compared to sea lions with papillomavirus infections, simultaneous infections and those with no apparent genital infections (GLM, $F_{3,36}$=2.73, p=0.05).

Supplementary material

Supplementary references:

Andersen CL, Jensen JL, Orntoft TF. 2004. Normalization of real-time quantitative reverse transcription-PCR data: a model-based variance estimation approach to identify genes suited for normalization, applied to bladder and colon cancer data sets. Cancer Res. 64:5245–5250.

Pfaffl MW, Tichopad A, Prgomet C, Neuvians TP. 2004. Determination of stable housekeeping genes, differentially regulated target genes and sample integrity: BestKeeper – Excel-based tool using pair-wise correlations. Biotechnol Lett. 26:509–515.

Schmittgen TD, Livak KJ. 2008. Analyzing real-time PCR data by the comparative CT method. Nat Protoc. 3:1101–1108.

Vandesompele J, De Preter K, Pattyn F, Poppe B, Van Roy N, De Paepe A, Speleman F. 2002. Accurate normalization of real-time quantitative RT-PCR data by geometric averaging of multiple internal control genes. Genome Biol. 3(7).

Vera-Massieu CA. 2015. Caracterización histológica y molecular de la respuesta inflamatoria durante el desarrollo temprano de crías de lobo marino de California, *Zalophus californianus*. MSc Thesis. Universidad Autónoma de Querétaro.
